# Tirofiban-Induced Profound Thrombocytopenia: A Case Report

**DOI:** 10.7759/cureus.82117

**Published:** 2025-04-11

**Authors:** Zeina Al Achkar, Robert Fakhoury, Osama F Zeindeen, Annoir Shayya

**Affiliations:** 1 Pulmonary and Critical Care Medicine, Lebanese American University Medical Center, Beirut, LBN; 2 Cardiology, Lebanese American University School of Medicine, Beirut, LBN; 3 Hematology/Oncology, Lebanese American University Medical Center, Beirut, LBN

**Keywords:** case report, drug-induced thrombocytopenia, platelets count, thrombocytopenia, tirofiban

## Abstract

Platelet glycoprotein IIB/IIIA antagonists are potent inhibitors of platelet aggregation. Although the incidence of thrombocytopenia following their administration is relatively low, it can sometimes lead to fatal consequences. We report a case of a 57-year-old gentleman presenting with non-ST elevation myocardial infarction complicated with tirofiban-induced profound thrombocytopenia that developed within 12 hours of administration. Given the high risk of acute-onset thrombocytopenia following treatment with glycoprotein IIb/IIIa inhibitors, platelet count monitoring is highly encouraged at frequent intervals. This case highlights the importance of early detection and treatment of thrombocytopenia.

## Introduction

Platelet glycoprotein IIB/IIIA antagonists are potent inhibitors of platelet aggregation; they block the common aggregation pathway and inhibit fibrinogen binding [1]. These drugs have been increasingly used in the treatment of acute coronary syndrome and percutaneous coronary interventions. Given the high bleeding risk associated with their use and the absence of improvement in outcomes in clinical trials, the American College of Cardiology (ACC) and the American Heart Association (AHA) recommended using them in patients with a large thrombus burden and in cases of slow or no-flow that are usually attributed to the presence of a distal embolus [2].

Although the incidence of thrombocytopenia following the administration of glycoprotein IIB/IIIA inhibitors is relatively low, ranging between 0.1% and 0.5%, the incidence of profound thrombocytopenia with a platelet count less than 2,000/mcL is only mentioned in a couple of case reports and can sometimes lead to fatal consequences. Tirofiban is one of the three glycoprotein IIB/IIIA inhibitors, and it is the only one available and used in Lebanon.

We report a case of a 57-year-old gentleman presenting with non-ST elevation myocardial infarction (NSTEMI) complicated with tirofiban-induced profound thrombocytopenia that developed within 12 hours of administration.

## Case presentation

A 57-year-old man presented to the emergency department with acute non-radiating sub-sternal chest pain associated with dyspnea at rest and desaturation (arterial oxygen saturation (SPO2) room air = 87%). His past medical history was significant for chronic kidney disease with a baseline creatinine of 1.6 milligrams per deciliter (mg/dL), hypertension, dyslipidemia, congestive heart failure, and a history of coronary artery disease with a previous coronary angiography in 2018 with implantation of three stents after which he was started on acetylsalicylic acid and clopidogrel. The patient was an ex-smoker with more than 20 pack/year history of smoking. Upon presentation to the emergency department, his baseline troponin level was 253 nanograms per liter (ng/L) (normal range < 34 ng/L), and the platelet count was 226,000 per microliter (mcL). An electrocardiogram (EKG) was done, and it was normal; no ischemic changes were found. Three hours later, his troponin level increased to 1,104 ng/L and then further to 2,237 ng/L. Moreover, the decision of coronary angiography was made despite the acute, on top of chronic, kidney disease, due to the worsening symptoms and rise in troponin.

**Figure 1:**
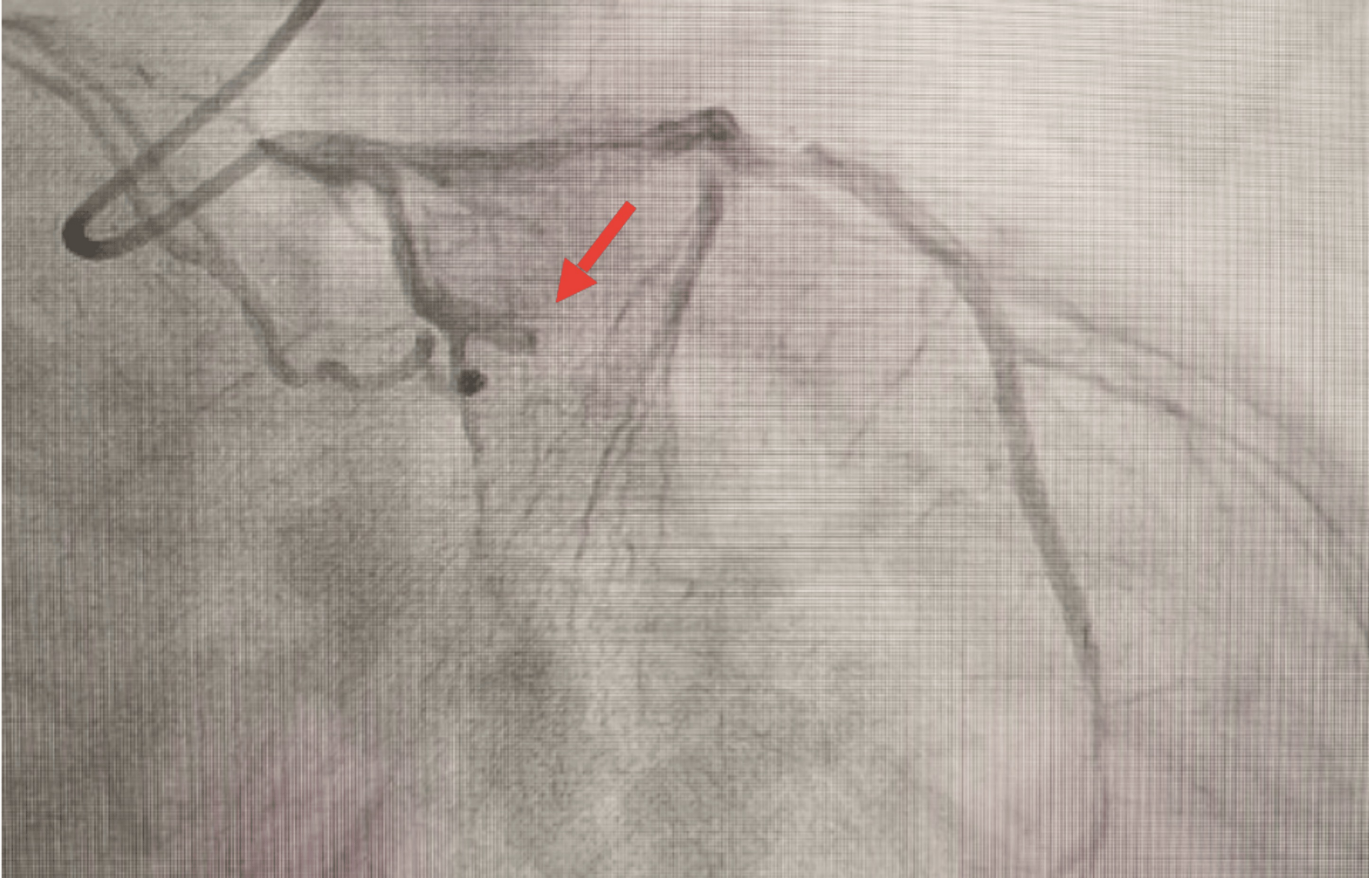
Complete obstruction of the marginal artery shown in invasive coronary angiography, highlighted by a red arrow

Coronary angiography showed an occlusion of the first marginal artery, as shown in Figure 1, where pre-dilatation was made with a balloon 2.0 × 15 mm, followed by stenting with a drug-eluting stent, given the patient's history of diabetes. Post-dilatation, Tirofiban was given along with 5,000 units of heparin secondary to distal embolization and the high clot burden. The patient was maintained on Tirofiban with renal-function-based dosing.

On the following day, 16 hours after the initiation of Tirofiban, the patient was found to have a severe drop in his platelet count, reaching 3,000/mcL. His physical examination was notable for purpura over bilateral lower extremities and scattered ecchymoses with no alarming features that were monitored closely. The patient had only minimal gingival bleeding otherwise and had no new cardiac or neurologic symptoms. Acetyl-salicylic acid, clopidogrel, enoxaparin, and tirofiban were discontinued immediately, and a peripheral smear was urgently performed, which showed no evidence of schistocytes or platelet aggregates. Repeat counts on the citrate tube showed similar results, confirming the profound thrombocytopenia. Platelet pheresis was requested to be prepared but not transfused due to the absence of serious bleeding and the fear of stent restenosis. Platelet factor 4 antibody was ordered and was negative, excluding the diagnosis of Heparin-induced thrombocytopenia in our clinical context. 

The complete blood count with differential (CBCD) was repeated six hours later, and it showed a further decrease in the platelet count, reaching 1,000/mcL, as shown in Table 1.

**Table 1:** Patient’s laboratory results

	Results on arrival	After 24 hours	After 30 hours	Normal range
Troponin	253 nanograms per liter (ng/L)	1,104 ng/L	2,237.9 ng/L	<34 ng/L
Platelets	226,000 per microliter (mcL)	3,000/mcL	1,000/mcL	150,000-400,000/mcL

Given the absence of any bleeding signs, the patient was given high doses of intravenous (IV) steroids, following which the platelet count started to increase gradually, reaching 22,000/mcL 24 hours later and returning to normal gradually. Antiplatelets were resumed on day 4. He received a total of three days of high-dose steroids.

On a further review of the patient’s history, he recalled a previous similar complication that followed his treatment with Tirofiban post-angioplasty six years ago, which was done at another hospital. Upon review of his chart, we found that angioplasty was done, followed by the insertion of three stents, one of them in the left anterior descending artery and two stents in the right coronary artery. On the day of angioplasty, the platelet count was 139,000/mcL, which was followed by a significant drop, reaching a minimum of 4,000/mcL the next day. Tirofiban was stopped immediately, and the patient received high doses of IV steroids, after which the platelet count started to improve gradually and returned to normal five days later.

## Discussion

Thrombocytopenia is generally divided into three stages according to its severity: mild when the platelet count is between 100,000/mcL and 150,000/mcL, moderate if between 50,000/mcL and 100,000/mcL, and severe if it is less than 50,000/mcL, with other references having it less than 20,000/mcL.

Tirofiban is a non-peptide molecule that binds to glycoprotein IIb/IIIa receptors on platelets, inhibiting their aggregation [3]. Five different presentations of thrombocytopenia following glycoprotein inhibitors administration have been described: 1) acute severe thrombocytopenia within 12 hours of first exposure (platelets <10 × 103/mcL), 2) acute thrombocytopenia within 12 hours of a second exposure, 3) delayed thrombocytopenia (five to seven days after treatment), 4) anaphylaxis after first or second exposure and 5) pseudo-thrombocytopenia [4]. Mild thrombocytopenia post-tirofiban occurred in around 1.1% to 1.9% of patients; however, severe thrombocytopenia occurred in 0.2% to 0.5 % [5]. In the real-world setting, profound thrombocytopenia is rarely encountered, resulting in scarce data regarding this matter. 

The exact mechanism behind thrombocytopenia post glycoprotein IIb/IIIa is not entirely well-demarcated. However, accumulated evidence showed that it might be mediated by drug-dependent antibodies [6-8]. A study conducted by Bougie et al. studying the etiology of acute severe thrombocytopenia in nine patients post tirofiban and eptifibatide treatment proved the presence of immunoglobulin G (IgG) antibodies by flow cytometry that reacted with the glycoprotein IIb/IIIa complex only with the presence of the medications [6]. Moreover, in this study, four patients were previously treated with one of these drugs. However, the rest were treatment-naïve, proving that these antibodies might not be only due to a previous drug exposure, but they can also naturally occur after the first exposure.

Pseudo-thrombocytopenia is an artifactual in vitro thrombocytopenia causing a falsely low platelet count [9]. Pseudo-thrombocytopenia, if treated as a true thrombocytopenia, by stopping anti-platelets and platelet transfusion, might increase the risk of thrombosis and transfusion reaction as well [4]. Peripheral smear is essential to differentiate between pseudo and true thrombocytopenia through the absence of platelet clumps [10]. Platelet count was repeated several times on citrated tubes in our case, excluding pseudo-thrombocytopenia.

In addition to the mentioned differentials, heparin-induced thrombocytopenia (HIT) is one of the serious pathologies to consider. HIT usually occurs after five to 10 days in patients with no previous exposure to heparin or after day one in those who received the drug in the past six months, which is not the clinical case with our patient. Furthermore, thrombocytopenia of moderate severity is the most common presentation in HIT, and developing severe thrombocytopenia is unusual [11]. The mainstay of HIT diagnosis is by measuring anti-platelet factor 4 antibodies, which was not ordered, given the low likelihood of it being the cause. In addition to that, there were no clinical signs by history or physical exam to point out thromboembolic events, which is a common feature in HIT.

Another possible cause of drug-induced thrombocytopenia is clopidogrel-associated thrombotic thrombocytopenic purpura (TTP), which often occurs within two weeks of treatment initiation and is fortunately uncommon [12]. TTP is characterized by micro-angiopathic hemolytic anemia, kidney injury, thrombocytopenia, fever, and neurologic manifestations. Our patient was already on clopidogrel for a long time, and the peripheral smear did not reveal the common findings, excluding this differential. Furthermore, the recovery of the platelet count following the resumption of Clopidogrel further attests to our diagnosis.

The cornerstone of the management of tirofiban-induced thrombocytopenia is based, most importantly, on stopping the medication immediately. Antiplatelets should be discontinued as well when the platelet count reaches 20,000/mcL or less or if there is any concomitant bleeding. Platelet transfusion is recommended whenever there is any serious active bleeding. However, the transfusion threshold in stable patients with no active bleeding is still controversial. Transfusing when the platelet count is less than 20,000/mcL is a common practice to prevent spontaneous bleeding [13]. Besides, some studies have shown that a lower threshold of <10,000/mcL can be safely tolerated in patients with no signs of bleed [14,15]. Moreover, intravenous immunoglobulin (IVIG) is one of the treatments that can be used; some reported cases showed improvement in the platelet count post-IVIG [16]. Other studies showed that there is no significant benefit from IVIG administration, and they advised combining it with steroids if used [5].

Given the high risk of acute-onset thrombocytopenia following treatment with glycoprotein IIb/IIIa inhibitors, platelet count monitoring is highly encouraged at frequent intervals, starting first at two to four hours post-infusion, followed by regular, frequent monitoring throughout the course of treatment. Recovery of platelet count starts immediately after the discontinuation of the offending medication; the usual time needed ranges from one to six days, with a mean of 2.1 days [17].

## Conclusions

Tirofiban-induced thrombocytopenia is a rare yet life-threatening complication. The acuity and seriousness of this side effect endorse the importance of early detection and treatment of thrombocytopenia and as such decrease the bleeding risk, as well as the risk of stent thrombosis and re-infarction.
